# Lessons from a multidisciplinary partnership involving women parliamentarians to address the overuse of caesarean section in Italy

**DOI:** 10.1136/bmjgh-2019-002025

**Published:** 2020-02-23

**Authors:** Pilar Montilla, Francesca Merzagora, Elisa Scolaro, Jennifer Requejo, Walter Ricciardi, Elena Meli, Adriana Bazzi, Giorgio Vittori, Flavia Bustreo, Rosanna Boldi, Maria Rizzoti, Mario Merialdi, Ana Pilar Betran

**Affiliations:** 1 Fondazione Cardiocentro Ticino, Lugano, Switzerland; 2 Osservatorio Nazionale sulla Salute della Donna e di Genere, Milan, Italy; 3 Reproductive Health and Research, World Health Organization, Geneva, Switzerland; 4 UNICEF USA, New York, New York, USA; 5 Catholic University of the Sacred Heart Rome, Rome, Italy; 6 Io Donna, Milan, Italy; 7 Corriere della Sera, Milan, Italy; 8 Ospedale San Carlo di Nancy, Rome, Italy; 9 Fondation Botnar, Basel, Switzerland; 10 Italian Chamber of Deputy, Rome, Italy; 11 Italian Senate of the Republic, Rome, Italy; 12 BD Global Health, Franklin Lakes, New Jersey, USA

**Keywords:** health policy, health systems, maternal health, obstetrics, public health

## Abstract

The increase of caesarean sections (CS) represents a global concern. Interventions tested to reduce unnecessary caesareans have shown limited success to date, partly because they have focused on medical perspectives or on single faceted interventions targeting only one group of stakeholders. Limited attention has been given to examining multidisciplinary and advocacy activities that could reduce unnecessary CS by raising awareness and engaging the media, advocacy groups, healthcare professionals and politicians. In 2009 in Italy, the national CS rate was the highest in Europe and momentum was building for action.

This case study includes a description of the activities conducted in Italy during 2009–2012 by a partnership that included the non-governmental organisation Osservatorio Nazionale sulla Salute della Donna, a bipartisan group of Italian women parliamentarians and the WHO. The objectives were to generate awareness about the increase and overuse of CS in Italy, to foster political actions to reverse this trend, to engage with the media and journalists and to better understand women’s birth preferences and needs.

A reduction of the CS rate has been observed in Italy following the activities of the initiative from 38.4% in 2009 to 34.2% in 2015 according to the Ministry of Health. Although we cannot infer a casual association between the Partnership and the CS decrease, it did contribute to political momentum and specific actions that should, in theory, have contributed to this reduction. These include the engagement of women parliamentarians for policy change, improved understanding of the local drivers of increases of CS including women’s needs and preferences, raising awareness and working with the media to convey appropriate information and an inclusive strategy giving the opportunity to local stakeholders to make their voices heard.

This partnership initiative illustrates a model for generating dialogue, reflection and action in countries showing signs of readiness to address escalating CS.

**Summary box**

- Multidisciplinary alliances and multifaceted interventions including advocacy activities are feasible and can play a crucial role in addressing complex challenges to the reduction of unnecessary caesarean sections.
- The momentum achieved in the Italian society to address high rates of caesarean sections allowed for the creation of a multidisciplinary partnership involving multiple stakeholders committed to action.
- Activities conducted by the Partnership from 2009 to 2012 were useful to generate awareness among all stakeholders about overutilisation of caesarean sections in Italy, to foster political actions to reverse this trend, to engage with the media for better information and to better understand women’s preferences and needs.
- In the years following the activities, the caesarean section rate in Italy decreased from 38.4% in 2009 to 34.2% in 2015 according to the Ministry of Health.
- Despite the multiple efforts being conducted worldwide to reduce unnecessary caesarean sections, documentation of these efforts and their impact is limited. Knowledge sharing through case studies and other approaches is essential for generating a sufficient evidence base on how best to reduce unnecessary caesarean sections across diverse settings.

## Introduction

Over the last few decades, the caesarean section (CS) rate has reached unprecedented levels worldwide, particularly in high and middle-income countries.^1^ Governments and clinicians have expressed concerns about this rise and the potential negative consequences for maternal and infant health.^2^ There are also financial consequences of the overutilisation of CS, as it requires more resources compared with vaginal deliveries. It has been estimated that the potential global savings if CS rates were to be reduced to 15% in those countries with rates above this percentage would be $2.32 billion.^3^

Despite global efforts to reverse the escalating trend, CS rates continue to increase and effective interventions to reduce unnecessary CS remain elusive.^4 5^ Multiple reasons underlie the limited success in stemming this trend with many factors and stakeholders involved.^6^ Women, families and healthcare providers are important players in decision-making about modes of delivery and they are all embedded within complex health systems and societies. Their views need to be considered as well as their preferences, the local culture and societal trends. The crucial importance of understanding the local context warrants undertaking case studies to explore examples of countries which have conducted efforts to understand and act on the overutilisation of CS by multifaceted interventions targeted at various levels of the health system, and at the wider societal level to promote attitudinal and behavioural changes.^6^

In 2009, the national CS rate in Italy had almost reached 40%, one of the highest rates of CS in Europe and a 260% increase since 1980 when the national average was 11%.^1 7^ We present in this manuscript the experience and activities of an initiative implemented in Italy from 2009 to 2012 to understand and try to reverse this trend. This country case study highlights the advocacy strategies used by a partnership (henceforth *the Partnership*) established by the non-governmental organisation Osservatorio Nazionale sulla Salute della Donna (ONDa—National Women’s Health Observatory, in English), a bipartisan group of Italian women parliamentarians and the Department of Reproductive Health and Research at the WHO, to create a supportive policy environment and political commitment for action to address the overutilisation of CS in Italy.

The objective of this partnership was to raise awareness on the overuse of CS and foster action to address this problem by involving women parliamentarians in political dialogue and action at national and subnational levels. Considering that the mass media is one of the most influential forces in contemporary culture and society, the initiative also involved collaboration with the media in order to disseminate appropriate messages about CS and vaginal delivery. In addition, to better understand women’s preferences and views on modes of delivery, a national survey was administered by *Io Donna*, one of the most important women’s magazines in Italy. This manuscript reports the activities, experience and lessons from this partnership in Italy.

## Caesarean section in Italy: rates, drivers and previous efforts to reduce escalating levels

**Figure 1:**
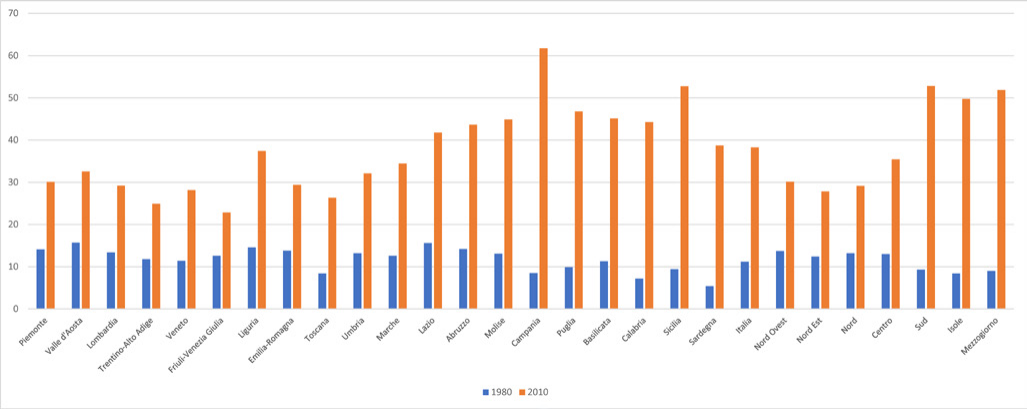
Caesarean section rates and trends in Italy by region (1980–2010). Source: National Institute of Statistics.^8^

Italy witnessed a steady increase in CS rates from 11.2% in 1980 to 23.2% in 1992, and 38.4% in 2009. Regional differences are also noticeable, ranging from 23% in Friuli-Venezia Giulia to 62% in Campania, with the highest rates occurring in Southern regions (figure 1).^8^

**Figure 2:**
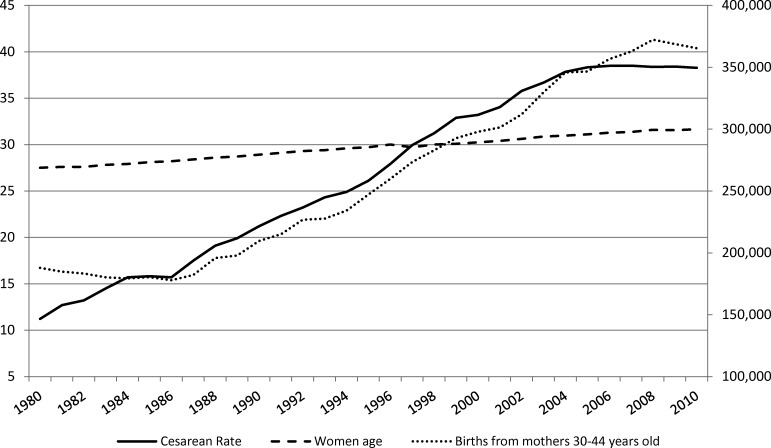
Trends in Italy, 1980–2010: caesarean section rate, average age of women at first child, number of births to women 30–44 years of age. Source: Data from Health for All Italia, Istituto Nazionale di Statistica (https://www.istat.it/it/archivio/14562).

Several non-clinical factors influence these regional variations including: maternal age, lack of access to epidural anaesthesia, fear of malpractice liability leading to practising defensive medicine, health service organisation structures and hospital economic incentives.^9^ An increasing proportion of women in Italy are delaying childbearing until after the age of 30 years, which is associated with higher risk and, consequently, higher CS rates.^10–12^ From 1980 to 2010 the proportion of births to women 30–44 years of age increased from 17% to 40% (figure 2), and the average age at first birth rose from 27 to 32 years. Limited access to analgesia may contribute to the high CS rate as fear of pain has been reported as a major reason for requesting a CS, especially among nulliparous and younger women.^12 13^ Even though most maternity units are equipped to provide epidural analgesia, access is not universal—epidural analgesia is used only in 16% of all vaginal deliveries nationwide^14^—since some units do not offer 24 hours’ service.^15^

**Figure 3:**
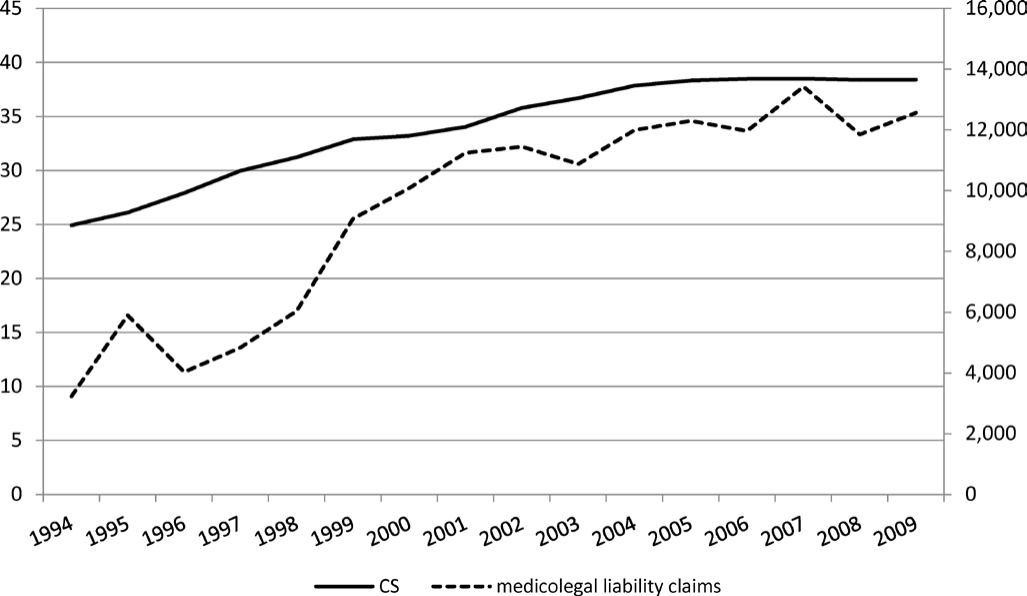
Trend in medicolegal claims in Italy and caesarean section (CS) rates. Source: Italian Chamber of Deputies (2011).^18^

Doctors are increasingly resorting to CS to avert the risk of malpractice suits.^16^ The number of legal proceedings related to medical malpractice has increased in Italy in the last decades. According to the National Association of Insurance Companies, claims for medical malpractice against physicians increased from about 3500 in 1994 to 12 559 in 2009 (figure 3). Hospitals spent over €10 billion (US$15.5 billion) to compensate patients injured through therapeutic and diagnostic errors.^17^ An investigation conducted by the Italian Parliament showed that, among 901 cases against healthcare professionals, 10% involved events occurring during pregnancy and labour.^18^ According to the Medical Union Federation (FESDEM), fetal distress, as a result of not having executed a CS, was the most common reason underlying the cases related to pregnancy and labour.^19^

Examining financial incentives and disincentives for hospitals provides additional insight into potential causes for increased utilisation of CS. Under the legislation at the time of the initiative, regional governments contracted public and private hospitals to deliver maternal health services. Each clinical procedure has a national rate, Tariffa Unica Convenzionale (TUC), to cover its costs. While the TUC for a vaginal delivery in 2010 was €1318, the TUC for a surgical delivery was €2457.^20^ Thus, increasing births by CS could increase hospitals’ revenues from reimbursement of services. However, every unnecessary CS implied an extra cost of €1139 for the Italian health system.

Prior to 2009, reduction in CS rates was set as a priority for the 2002–2004 National Health Plan establishing guidelines for indications for elective CS, recommendations on the appropriateness of diagnostic procedures and manoeuvres used in clinical practice and considerations on the implications of a CS in subsequent pregnancies.^21^ However, to our knowledge the project described in this manuscript is the first multisectorial partnership initiative created and implemented in Italy to address the increase of CS use, to generate awareness and to foster political actions to reverse this trend.

## Activities of the initiative

In 2009, WHO established the Partnership with ONDa and a group of bipartisan parliamentarians with the aim to address the overuse of CS in Italy. Given the complexity of the challenge, the Partnership extended the debate beyond public health and clinical guidance and started an inclusive policy dialogue embracing the cultural, social, political and economic dimensions. In addition, it was considered crucial to engage all stakeholders including institutions, policymakers, health professionals, media and relevant citizens. Table 1 shows the stakeholders engaged in the initiative by level (national, regional or community) as well as the relevant key activities and actions at each level.

**Table 1:** Stakeholders engaged in the initiative by level (national, regional or community) and relevant key activities and actions at each level

Level	Target	Key advocacy issues
National level	National health, policymakers, legislators, regulators	Assessing progress in maternal and infant health. Monitoring emergency obstetric care and resource use. Allocating resources to maternal health programmes at the country level. Implementing policy to improve maternal health quality services.
Regional level	Health programme managers	Allocating funds to increase availability of epidural anaesthesia during labour at all the birth centres. Building training initiatives for healthcare operators to work under ‘Code Red’ situations during labour and delivery. Adopting a caesarean section classification system to monitor and compare caesarean section rates in a consistent and action-oriented manner.
	Clinicians attending women: family doctors, gynaecologists, midwives	Educating patients about the risks of caesareans and promotion of vaginal delivery as a natural event. Training.
	Community leaders and maternal health advocates	Disseminating information about delivery methods. Creating advocacy agenda and the use of small and mass media and interpersonal communications to influence perceptions about vaginal delivery. Mobilising women to demand access to maternal health services with high-quality standards.
Community level	Media	Ensuring communities are informed regarding the pros and cons of CS. Creating awareness about the overutilisation of CS.
	Women	Keeping informed and making choices according to their health needs.

CS, caesarean section.

It was expected that the Partnership would (1) increase awareness among parliamentarians, policymakers and institutions on this issue; (2) stimulate political action at national and regional levels; (3) collaborate with the media to create awareness at the public level; and (4) lead efforts for a better understanding of women’s preferences and views on mode of delivery. During the period 2009–2012, the Partnership was able to accomplish its goals by implementing a series of activities which are described below.

### Technical meetings to increase awareness among parliamentarians

A technical meeting was held at WHO Headquarters in Geneva in January 2009. The objective of the meeting was to raise awareness and improve understanding on risks and benefits of vaginal birth and CS, the steady increase of unnecessary CS in Italy, causes of excessive use of CS and the potential solutions. Eight parliamentarians representing the major political parties attended the meeting. Journalists from the Italian national newspapers were invited to cover the event. Journalists’ participation ensured the consideration of sociocultural issues and the dissemination of the key outcomes of the meeting in the major Italian newspapers. Participants were briefed by WHO and the president of the Italian Society of Gynecology and Obstetrics (Societá Italiana di Ginecologia e Ostetricia, SIGO).

Three follow-up technical meetings were organised on a yearly basis to monitor progress and plan for new action as the commitment for political action extended to other groups of parliamentarians.

### Political action for legislation and policy

The technical meeting concluded with the agreement to present the issue of overuse of CS to the Italian Parliament as a parliamentary motion. A motion is a proposal approved by the parliament calling for legislative and political action at the national and regional governments. The eight parliamentarians, based on the information received at the technical meeting at WHO, took responsibility for drafting and presenting the parliamentarian motion to the parliament. The motion was presented by Senator Laura Bianconi on 2 April 2009 (sitting No 187) and subsequently approved by the Senate on 10 June 2009 (sitting No 219).^22^

The approved motion represented a commitment of the government to take specific action to address overutilisation of CS. The parliamentary motion included the following specific recommendations: implementation of clinical guidelines for the decision of CS; development of instruments to analyse CS trends on a continuous basis; allocation of increased resources at the regional level to provide free epidural analgesia; promulgation of legislation and norms aimed at reducing the practice of ‘defensive medicine’; and provision of appropriate, accurate and useful information to women about birth including the risks and benefits of each mode of birth.^22^ Two additional motions were prepared subsequently involving the engagement of more parliamentarians.^23 24^ All these new proposals were discussed at the Senate Assembly on 10 June 2009 (sitting No 219) and resulted in the achievements depicted in table 2.

**Table 2:** Results of the political engagement and commitment of parliamentarians and recommendations from the local round tables

Political commitment achievements	Recommendations from local round tables
Political support to promote the agreement between the national government and the regions to implement an action plan for the promotion and improvement of quality, safety and appropriateness of care interventions in the birthing process and for the reduction of caesarean section. Participation on the parliamentary hearings on ‘Safe birth’ (Nascere sicuri) that were held from September 2010 to November 2012. The hearings addressed key issues regarding the quality of the birthing process in Italy and the situation of the birthing/childbirth centres and led to the investigation of the lack of self-determination of women in the choice for mode of birth.^15^ Adoption of the ministerial CS guidelines at subnational level which promotes discussion between women and providers about mode of delivery, and risks and benefits when women request a CS which is not medically indicated as well as documentation of the entire decision-making path in the medical record. In the absence of an appropriate clinical indication, the doctor has the right to refuse a request for a planned caesarean section.^21^	Developing and implementing national guidelines on CS. Facilitating and promoting doctor–patient discussions on the mode of delivery. Providing training and clinical updates for physicians, midwives and nurses to handle emergency situations during labour and delivery. Implementation of best practice approaches to manage women with previous CS. Improving access to epidural analgesia in all hospitals. Providing efficient audit instruments to monitor CS rates, such as nationwide use of the Robson classification as a standard system. Providing more information to women on mode of delivery.

CS, caesarean section.

### Engaging stakeholders at the subnational level

The parliamentarians also implemented activities in selected regions by organising regional round tables for in-depth analysis of local trends in CS rates and drivers, economic implications and specific actions undertaken by regional governments. To account for regional variations in CS rates, technical round tables in 2010 were held in three regions: Campania (61% CS rate), Lombardia (29% CS rate) and Emilia Romagna (30% CS rate). In order to assess and analyse regional stakeholders’ perceptions, representatives of the regional health authority, medical schools, health professional associations, hospitals and journalists were invited. These round tables were supported technically by the WHO and the SIGO. Participants discussed their views on local statistics and information, clinical models, and perceived challenges and barriers to reverse the CS trends in their region.

The discussions in the round tables offered the opportunity for local stakeholders to express their views and opinions and to make their voice heard at the national level. They provided important information on healthcare providers’ needs and challenges within the Italian health system. After every round table, a short summary document was prepared for the media. It was also uploaded on ONDa’s website.^25^ A summary of the recommendations is shown in table 2.

### Collaboration with the media for accurate information dissemination

A collaboration with the media resulted in increased visibility of the Partnership’s activities and increased interest and debate in the media over mode of delivery. During the years of the initiative, the Partnership was committed to the provision of clear, evidence-based and accurate information to the media in a timely manner. A standard format for the communication to the press was agreed with journalists which was consistently used and improved understanding and communication with the media. During 2009–2011 more than 200 articles were published in women’s magazines in Italy (eg, Io Donna, Donna Moderna, Vanity Fair) which represent an important source of information on a variety of topics including pregnancy and childbirth and may influence women’s opinion.^26 27^ Local TV talk shows discussed this issue with the participation of women and local policymakers and news programmes to generate interest in and support for vaginal delivery.

### Addressing women’s needs for information and understanding preferences on mode of delivery

The Partnership sponsored the production of a 30 seconds commercial spot, endorsed by the Ministry of Equal Opportunities and broadcast on several television channels (http://www.ondaosservatorio.it/spot-sul-parto-cesareo/). The spot was designed and shot by a professional marketing and advertising company pro bono. The commercial spot captured the need of a CS as a surgical life-saving procedure in certain situations, but also emphasised that for the majority of women, giving birth would be a natural event without the need of the surgical procedure. The spot was broadcast in a private channel of television (Sky) three times a day during 4 days of April 2010, and in the main public channels (RAI 1, RAI 2 and RAI 3) three times a day for 15 days during September 2010. On an average day, RAI had a relatively high television audience share of 41.3% in 2010.^28^ These television channels aired the spot for free.

Simultaneously, the Partnership established a collaboration with Io Donna, one of the most popular women’s magazines in Italy. Three activities were the focus of this collaboration: featuring articles related to birth in the magazines, the release of a brochure on mode of birth and administering the first national survey on preferences for mode of delivery and reasons among Italian women.

To address the common misconception that CS is safer than vaginal delivery, a brochure targeting women was produced in the second semester 2009 providing information on when and why to have a CS and explaining its advantages and disadvantages. This brochure was uploaded at ONDa’s website, distributed by Io Donna and through public hospitals and clinics.

A cross-sectional survey on preferences for mode of delivery was administered by Io Donna in both the printed issue in December 2010 and the web page. The sample size was 1000 women leaving in Italy in two phases: (1) 250 recruited through the web page of the woman’s magazine; and (2) 750 through telephone interviews using anonymous structure questionnaires. The results of the survey have been published elsewhere^12^ and showed that, despite the high national CS rate, 80% of women who responded declared they would prefer to deliver vaginally. Reasons cited for this preference were the immediate contact with the newborn, a shorter hospital stay and a faster postpartum recovery. On the other hand, the main reasons for preferring a CS were fear of pain, convenience to schedule the delivery and because it was perceived as being less traumatic for the baby. In this survey, obstetricians were considered as the source which most influenced Italian women’s preferences.^12^

A press conference was held in September 2011 to release the findings of the survey. Experts and policymakers were invited to comment on the survey’s results during the conference. A press kit was distributed to journalists with a news release, fact sheets about high CS rates, public health implications and WHO relevant documents and guidelines.

## Lessons learnt

A reduction of CS rate has been observed in the years following the activities of the initiative from the 38.4% in 2009 to 34.2% in 2015 according to the latest Ministry of Health’s report on birth,^29^ or to 33.6% in 2017 according to the National Observatory on the Regional Health by the Public Health Institute.^30^ We cannot infer a casual association between the activities rolled out by the initiative and the decrease, but we believe these actions could have contributed to the trend.

We are not aware of other concurrent activities or programmes that could be responsible for this reduction. To our knowledge, there are no other campaigns of this nature and magnitude having been conducted in Italy.

### Drivers contributing to the achievements

We believe that this country case study identifies guiding principles and crucial elements contributing to the achievement of the Partnership’s goals. The engagement of women parliamentarians was a key strategy in influencing national and regional authorities to address the overuse of CS in Italy and leading legislative processes. However, identifying the parliamentarians more receptive to maternal health-related topics was crucial for a sustained action. In this initiative, local non-profit organisations such as ONDa, with missions oriented towards improving health, can play a strategic role in guiding parliamentarians and building bridges among national and regional institutions and healthcare operators. Building on previous experience was crucial to appropriately frame the challenge. All partners involved had previous experience and an in-depth understanding of the topic from different perspectives and sectors. The inclusion of subnational health system levels provided local stakeholders the opportunity for voicing their concerns and recommendations and revealed the complex, dynamic and somehow context-specific drivers of CS. All these factors are essential to consider in the design and implementation of strategies to reduce unnecessary CS and ultimately to promote country ownership.

The collaboration with the media catalysed the initiative’s effort for political and social awareness in the overuse of CS, its risks and effects in the health system. Implementing the survey in Io Donna channelled women’s voices, helped understand women’s opinions and preferences on mode of delivery and identified misunderstandings about the causes of a problem.

### Challenges and opportunities for improvement

Challenges encountered or potentially envisioned in other settings attempting to replicate this initiative are presented in table 3 as well as recommendations to overcome these challenges.

**Table 3:** Challenges and recommendations to overcome these challenges

Challenge	Recommendations to overcome the challenge
Identification of parliamentarians	Engage strong organisations (eg, NGO) with missions oriented towards improving health and with interaction with political entities. These local partners can facilitate the engagement of parliamentarians who may be more receptive and possibly with previous engagements on these issues so that previous experiences and knowledge are taken into account.
Identification of groups and individuals to support the activities	Engage strong local NGOs with capacity to build sound strategic relationships with local groups and individuals who can be persuaded to support in advocacy efforts.
Operationalisation of the collaboration	The selection of the constituencies is crucial for sustained effort. Local organisations are able to identify constituencies with previous experience or engaged in these issues.
Design an appropriate and culturally acceptable framework to position caesarean section	Even though the reduction of caesarean section was already set as a priority in Italy, it is crucial to design an advocacy framework in a positive light with a positive message that cannot be misunderstood. Ensure that the use of caesarean section when medically necessary is not stigmatised and establish the focus on the reduction of the overuse, that is, the non-medically necessary caesarean section.
Design of activities	The inclusion of partners with complementary expertise is highly recommended. WHO identified the unmet need and provided the technical and scientific information while the strengths of other partners were directed towards the design of the interventions and activities. Based on experience and local capacity, these activities need to be locally relevant and culturally acceptable.
Engaging the media	Ensure the participation of the journalists in technical meetings in which issues such as the overuse of caesarean section, risks and benefits of each mode of birth and the effects in the women, children and health systems are analysed. Sensitising and ‘educating’ the media is key. Engage communication experts with interest in the topic who are able to frame public health issues with tailored messages that connect and resonate with the public and with the society as a whole.

NGO, non-governmental organisation.

Opportunities for improvement were also identified. A more intense or frequent follow-up with the partners and, in particular, with the parliamentarians and the journalists, could have resulted in a more powerful longer term action at all levels. Although this initiative was not implemented as a research activity with a built-in monitoring component, more organised or structured monitoring activities (eg, regular assessment of process or outcomes, achievements, barriers encountered) may have provided key information to redirect action when and if necessary (eg, monitoring legislation at regional level and provision of updates as relevant local policies were approved). Similarly, we did not implement activities to measure the impact of the media activities such as the commercial spot on knowledge acquisition or behaviour change. Stronger emphasis on the dissemination of the survey results could have increased awareness of the risks of unnecessary caesarean sections and benefits of vaginal delivery more significantly.

## Conclusions

The Partnership initiative described in this manuscript offered an opportunity for dialogue, reflection and action from a political, medical and social perspective, in a country showing signs of readiness to address this issue. The advocacy activities developed by the Partnership WHO-Parliamentarians-ONDa during 2009–2012 facilitated several policy changes at national and regional levels. Based on the experience of this country, we have proposed diverse actions that government, parliamentarians and advocates could implement to increase awareness about the overuse of CS and to propose relevant actions to reduce unnecessary CS.

Finally, given the scarce research studies on strategies for reducing overuse of CS, it is important to document successful stories and multidisciplinary collaborative and comprehensive approaches in raising awareness that may prove useful for other countries as they work towards a rational use of CS.
